# Effects of Curcumin on Oxidative Stress and Ferroptosis in Acute Ammonia Stress-Induced Liver Injury in Gibel Carp (*Carassius gibelio*)

**DOI:** 10.3390/ijms24076441

**Published:** 2023-03-29

**Authors:** Liyun Wu, Bo Dong, Qiaozhen Chen, Yu Wang, Dong Han, Xiaoming Zhu, Haokun Liu, Zhimin Zhang, Yunxia Yang, Shouqi Xie, Junyan Jin

**Affiliations:** 1 State Key Laboratory of Freshwater Ecology and Biotechnology, Institute of Hydrobiology, Chinese Academy of Sciences, Wuhan 430072, China; 2 University of Chinese Academy of Sciences, Beijing 100049, China; 3 The Innovative Academy of Seed Design, Chinese Academy of Sciences, Beijing 100101, China

**Keywords:** ammonia, curcumin, ferroptosis, oxidative stress, liver injury

## Abstract

This study investigated the potential role of curcumin (CUR) in preventing oxidative stress and ferroptosis induced by ammonia exposure in gibel carp. Experimental fish (initial weight: 11.22 ± 0.10 g, *n* = 150) were fed diets supplemented with or without 0.5% CUR for 56 days, followed by a 24 h ammonia (32.5 mg/L) exposure. Liver damages (aspartate aminotransferase (AST), alanine aminotransferase (ALT), adenosine deaminase (ADA), and alkaline phosphatase (ALP)) and oxidative stress enzyme activities (reactive oxygen species (ROS), malondialdehyde (MDA); and the content of antioxidant capacity (T-AOC), superoxide dismutase (SOD), and glutathione peroxidase (GPx)) were induced by ammonia stress. The antioxidant capacity was decreased, as indicated by inhibited gene expression of nuclear factor erythroid 2-related factor 2 (*nrf2*), heme oxygenase-1 (*ho-1*), catalase (*cat*), and *sod*. Ferroptosis was induced by ammonia stress, as suggested by upregulated mRNA levels of nuclear receptor coactivator 4 (*ncoa4*), transferrin receptor 1 (*tfr1*), and iron-responsive element-binding protein 2 (*ireb2*), and downregulated expression of glutathione peroxidase 4 (*gpx4*), ferroportin (*fpn*), and ferritin heavy chain 1 (*fth1*). In addition, both mRNA and protein levels of ferroptosis markers acyl-CoA synthetase long-chain family member 4 (ACSL4) and prostaglandin-endoperoxide synthase 2 (PTGS2) were upregulated, while cystine/glutamate antiporter (SLC7A11) was downregulated. However, liver injury and ferroptosis in fish induced by ammonia could be attenuated by CUR. Collectively, these findings demonstrate that CUR ameliorates oxidative stress and attenuates ammonia stress-induced ferroptosis. This study provides a new perspective on potential preventive strategies against ammonia stress in gibel carp by dietary CUR.

## 1. Introduction

Ammonia is the most common toxic substance in the aquaculture environment. In recent years, the intensification and diversification of aquaculture globally have been continuously increasing. Consequently, large amounts of ammonia in the water are produced by unconsumed feed as well as through the feces of aquatic animals. High concentrations of ammonia are toxic to fish, as ammonia can produce excessive accumulation of reactive oxygen species (ROS), thereby inducing oxidative stress in fish and causing an imbalance in the metabolic system, including damage to liver function, and eventually leading to massive fish mortality [1]. As an essential organ for detoxification, the liver is highly susceptible to ammonia toxicity [2]. Increasing the activity of antioxidant enzymes, such as superoxide dismutase (SOD) and glutathione peroxidase (GPx), is one of the most important defense strategies to eliminate ROS in fish [3]. Plasma levels of aspartate aminotransferase (AST) and alanine aminotransferase (ALT) are considered sensitive biological indicators for assessing liver damage in fish due to environmental stress [4]. In common carp (*Cyprinus carpio*), SOD, GPx, AST, and ALT levels were increased by ammonia exposure [5]. Nuclear factor erythroid 2-related factor 2 (Nrf2), a key transcription factor in regulating genes associated with oxidative damage, was also induced by ammonia [6,7].

Ferroptosis is a novel and unique form of programmed cell death, and it is distinctly different from apoptosis, necrosis, autophagy, or other types of cell death at the morphological, biological, and genetic levels [8,9]. ROS accumulation and lipid peroxidation are the central pathways triggering ferroptosis [10,11]. Acyl-CoA synthetase long-chain family member 4 (ACSL4) is involved in fatty acid metabolism that subsequently contributes to lipid peroxidation, and it has been suggested as a potential biomarker and driver of ferroptosis [12]. Inhibiting the expression of cystine/glutamate antiporter (SLC7A11) can induce ferroptosis by damaging the antioxidant capacity of cells [13]. Prostaglandin-endoperoxide synthase 2 (PTGS2) is considered a marker of ferroptosis [14]. It has been reported that inhibition of ferroptosis by reducing the formation of lipid peroxidation products in the body through nutritional pathways may provide a potential protective strategy against related pathologies [15]. At present, it is unclear whether ammonia stress induces hepatocyte death through induced ferroptosis.

Curcumin (CUR) is an orange-yellow polyphenolic substance extracted from the ginger family that has potent antioxidant, bactericidal, anti-inflammatory, and hepatobiliary protective effects [16]. CUR has been shown to inhibit deltamethrin-induced oxidative stress, inflammation, and apoptosis in Northern snakehead (*Channa argus*) by regulating the Nrf2 signaling pathway [7]. CUR attenuated aflatoxin toxic B1-induced liver injury via mediating ferroptosis and the Nrf2 pathway in mice [17]. Thus, CUR could potentially alleviate these responses by regulating ferroptosis and oxidative stress.

Gibel carp (*Carassius gibelio*) is an important economic freshwater fish. The yield reached 2748.6 thousand tonnes in 2020 in the world [18]. Ammonia toxicity has been the main cause of growth inhibition, oxidative stress, and reduced immunity in crucian carp (*Carassius auratus*) [19]. It is important to attenuate stress induced by ammonia in aquaculture. We hypothesized that CUR could regulate oxidative stress and ferroptosis to ameliorate the adverse effects of acute ammonia exposure on gibel carp. To test our hypothesis, we stimulated the gibel carp via ammonia exposure and applied CUR to intervene, primarily to elucidate the expression of oxidative stress and ferroptosis markers PTGS2, ACSL4, and SLC7A11 before and after CUR administration.

## 2. Results

### 2.1. Growth Performance and Body Composition

As shown in Table 1, dietary supplementation with CUR significantly increased feeding rate (FR) (*p* < 0.05), but there was no significant effect on feed efficiency (FE) or specific growth rate (SGR) of gibel carp (*p* > 0.05). No significant differences were observed in whole-body crude protein, crude lipid, or moisture, while ash content was significantly different between the two groups (*p* < 0.05).

### 2.2. Plasma and Liver Metabolites

**Figure 1:**
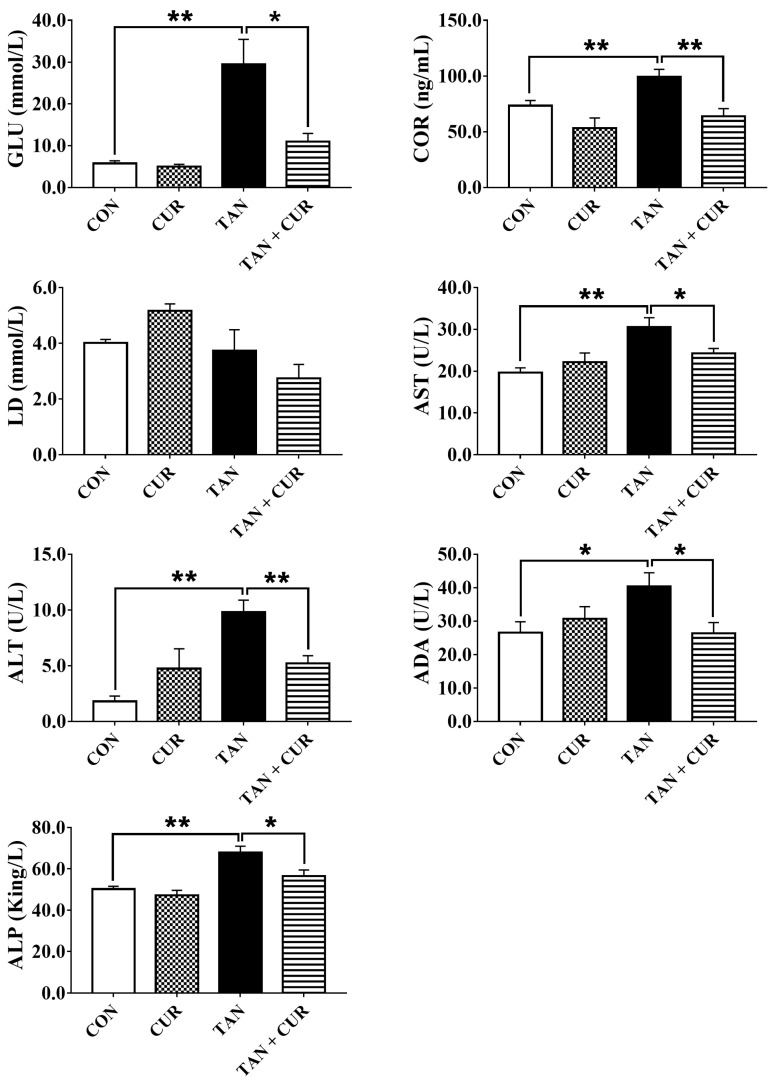
The changes in plasma metabolites in gibel carp after eight weeks of feeding CUR diets followed by acute ammonia stress. CON—control; CUR—curcumin; TAN—total ammonia nitrogen; TAN + CUR—total ammonia nitrogen + curcumin. GLU—glucose; COR—cortisol; LD—lactic acid; AST—aspartate transaminase; ALT—alanine aminotransferase; ADA—adenosine deaminase; ALP—alkaline phosphatase. The values are expressed as the mean ± standard error (*n* = 6). * *p* < 0.05; ** *p* < 0.01.

**Figure 2:**
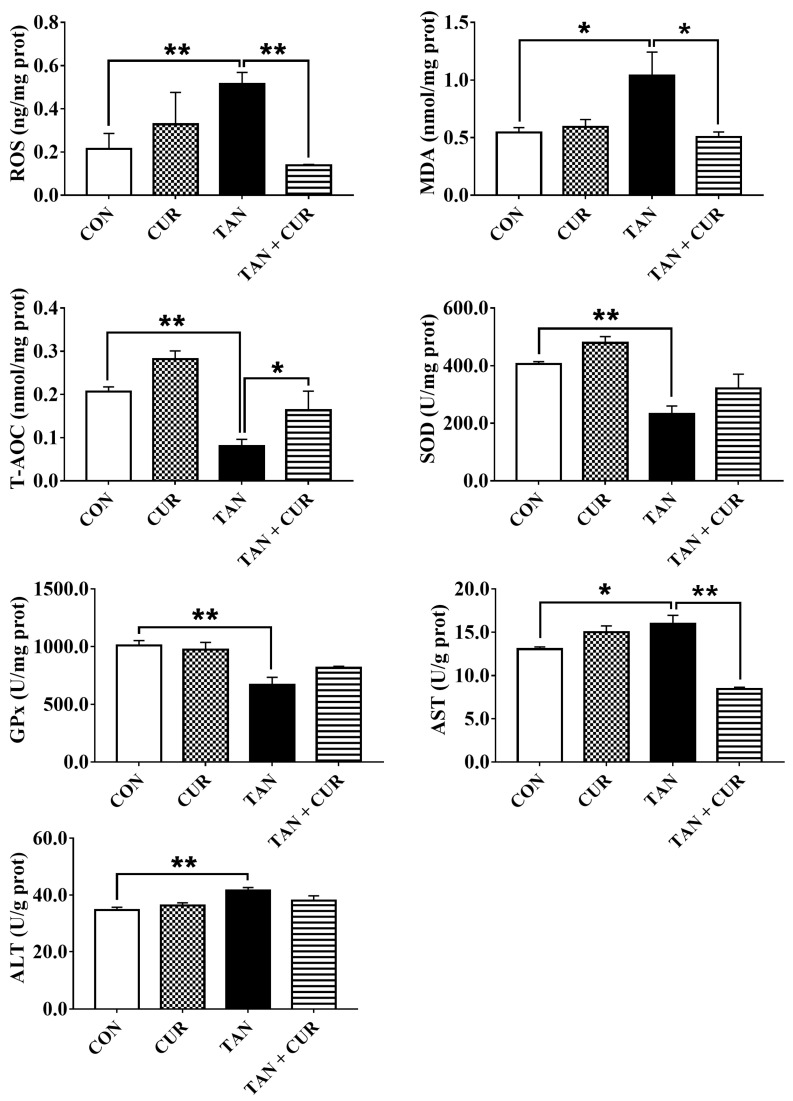
Changes in liver metabolites in gibel carp after eight weeks of feeding CUR diets followed by acute ammonia stress. CON—control; CUR—curcumin; TAN—total ammonia nitrogen; TAN + CUR—total ammonia nitrogen + curcumin. ROS—reactive oxygen species; MDA—malondialdehyde; T-AOC—total antioxidant capacity; SOD—superoxide dismutase; GPx—glutathione peroxidase. The values are expressed as the mean ± standard error (*n* = 6). * *p* < 0.05; ** *p* < 0.01.

After ammonia exposure, plasma glucose (GLU), cortisol (COR), aspartate aminotransferase (AST), alanine aminotransferase (ALT), adenosine deaminase (ADA), and alkaline phosphatase (ALP) levels were significantly increased in the control (CON) group (Table S1). However, the addition of CUR significantly reduced the levels of the abovementioned indexes. No significant difference was found in plasma lactic acid (LD) content among the treatment groups (Figure 1). As shown in Figure 2, liver ROS, malondialdehyde (MDA), AST, and ALT contents in the CON group were significantly increased by ammonia treatment, while antioxidant capacity (T-AOC), SOD, and GPx activities were significantly decreased (Table S1). In addition, compared with the total ammonia nitrogen (TAN) group, the total ammonia nitrogen + curcumin (TAN + CUR) group significantly had decreased ROS, MDA, and AST contents and increased T-AOC. No changes were found in SOD, GPx, or ALT activities (Table S1).

### 2.3. Tissue Histopathology

**Figure 3:**
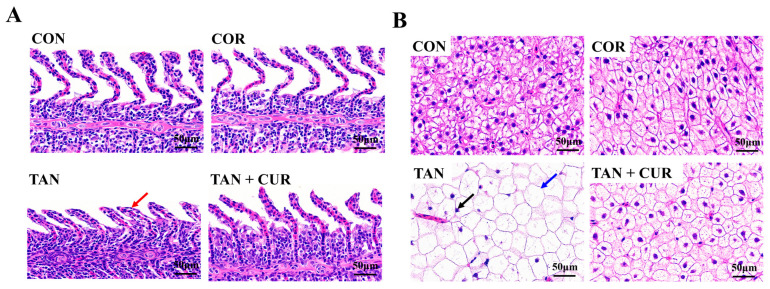
After feeding CUR diets for eight weeks, the histopathological damage caused by acute ammonia stress to gibel carp was improved. (**A**) Histological images of gills in gibel carp. (**B**) Histological images of liver in gibel carp (red arrows indicate atrophy of gill lamellae; black arrows indicate nucleus deviation; blue arrows indicate swelling of hepatocytes as well as vacuolization). CON—control; CUR—curcumin; TAN—total ammonia nitrogen; TAN + CUR—total ammonia nitrogen + curcumin. Scale bar = 50 μm.

The gill histological results showed that the gill lamellae in the CON, CUR, and TAN + CUR groups were in significantly better condition than those in the TAN group (Figure 3A), and the gill tissue structure in the first three groups was intact, with a large number of gill lamellae neatly and tightly arranged on the gill filaments and without visible damage. However, the branchial tissue of the TAN group showed pathological changes, with partial branchial hypotrophy and flattening and shortening in the visual field. As shown in Figure 3B, the liver histology in the CON and CUR groups was basically normal. By contrast, the liver tissue of the TAN group showed abnormalities, some hepatocytes were slightly hypertrophic, with the nucleus disappearing or fragmenting, and the cytoplasm was vacuolated. The liver injury in the TAN + CUR group was effectively relieved after ingesting CUR feed.

### 2.4. Nuclear Factor [Erythroid-Derived 2]-like 2 (Nrf2) Signaling Pathway

**Figure 4:**
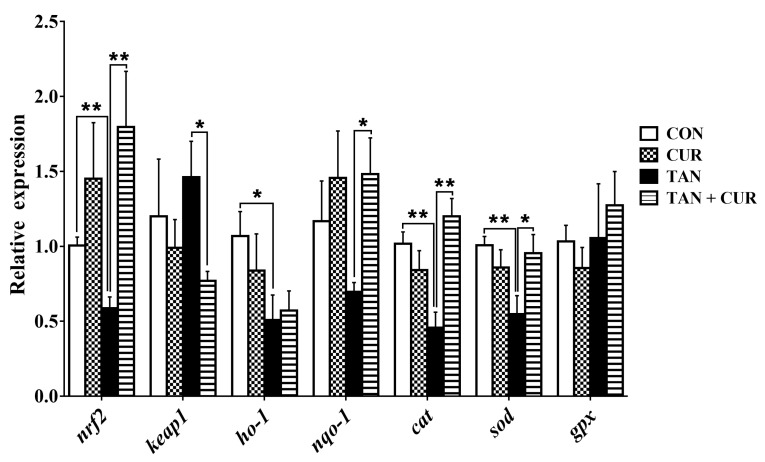
The mRNA levels of genes involved in the nuclear factor [erythroid-derived 2]-like 2 (Nrf2) signaling pathway in the normal and acute ammonia stress groups of gibel carp after feeding CUR diets for eight weeks. CON—control; CUR—curcumin; TAN—total ammonia nitrogen; TAN + CUR—total ammonia nitrogen + curcumin. *keap1*—kelch-like ECH-associated protein-1; *ho-1*—heme oxygenase-1; *nqo-1*—NADPH quinine oxidoreductase-1; *cat*—catalase. The values are expressed as the mean ± standard error (*n* = 6). * *p* < 0.05; ** *p* < 0.01.

In gibel carp, the mRNA levels of *nrf2*, heme oxygenase-1 (*ho-1*), catalase (*cat*), and *sod* were significantly decreased in the TAN group compared with the CON group, and the mRNA levels of kelch-like ECH-associated protein-1 (*keap1*) and NADPH quinine oxidoreductase-1 (*nqo-1*) were unaltered (Figure 4). After ingestion of the CUR diet, mRNA levels of *nrf2*, *nqo-1*, *cat*, and *sod* were significantly increased in the TAN + CUR group. There was no statistically significant effect on the mRNA levels of *gpx* regardless of the treatment.

### 2.5. Ferroptosis

**Figure 5:**
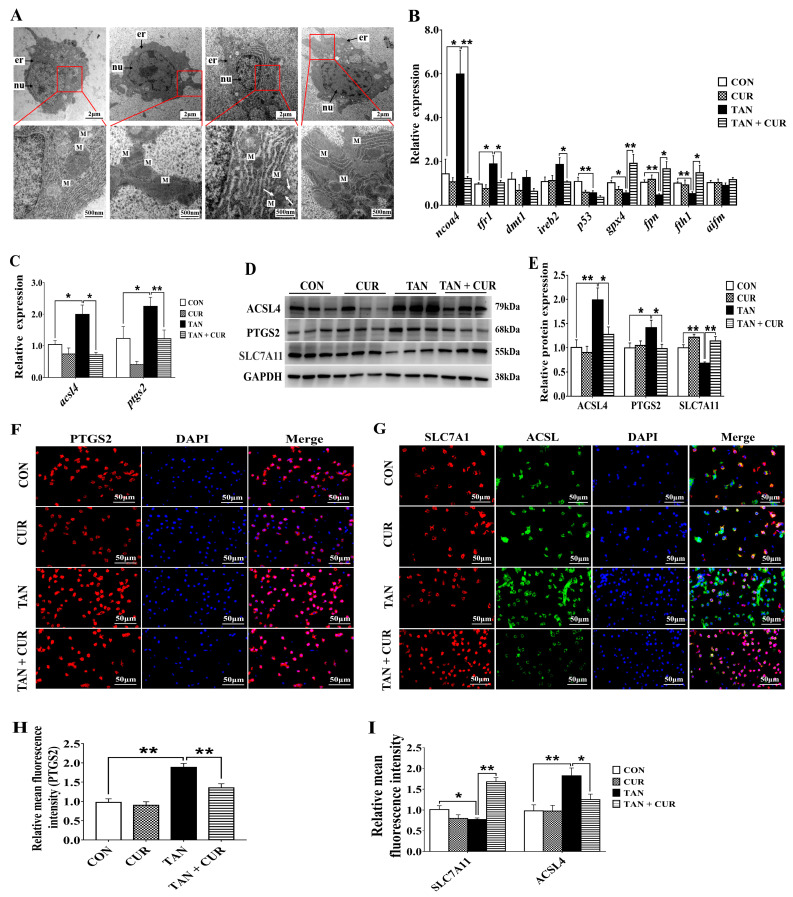
The ferroptosis caused by acute ammonia stress was reduced in gibel carp after feeding CUR diet for eight weeks. CON—control; CUR—curcumin; TAN—total ammonia nitrogen; TAN + CUR—total ammonia nitrogen + curcumin. (**A**) Ultrastructure (Transmission Electron Microscopy (TEM); the white arrow points to the rupture of the mitochondrial outer membrane); M—mitochondria; nu—nucleus; er—endoplasmic reticulum. (**B**) mRNA levels of ferroptosis-related genes. *ncoa4*—nuclear receptor coactivator 4; *tfr1*—transferrin receptor 1; *dmt1*—divalent metal transporter 1; *ireb2*—iron-responsive element-binding protein 2; *p53*—cellular tumor antigen; *gpx4*—glutathione peroxidase 4; *fpn*—ferroportin; *fth1*—ferritin heavy chain 1; *aifm2*—apoptosis-inducing factor mitochondria-associated 2. (**C**) mRNA levels of acsl4 and ptgs2. *ptgs2*—prostaglandin-endoperoxide synthase 2; *acsl4*—long chain acyl CoA synthetase 4. (**D**) Ferroptosis key protein expression. (**E**) Relative strength of proteins involved in ferroptosis. (**F**) Fluorescence intensity of PTGS2 (red colour) by immunofluorescence assay. Nuclei were stained with DAPI (blue color). scale bar = 50 μm. (**G**) Fluorescence intensity of SLC7A11 (red color) and ACSL4 (green color) by immunofluorescence assay. Nuclei were stained with DAPI (blue color). scale bar = 50 μm. (**H**) Quantification of PTGS2 fluorescence intensity. (**I**) Quantification of SLC7A11 and ACSL4 fluorescence intensity. The values are expressed as the mean ± standard error (*n* = 6). * *p* < 0.05; ** *p* < 0.01.

Transmission Electron Microscopy (TEM) results showed that compared with the CON group, the liver tissue in the TAN group exhibited mitochondrial outer membrane rupture and mitochondrial atrophy accompanied by reduction or disappearance of mitochondrial ridges and increased membrane density. After CUR treatment, the mitochondrial morphology was significantly improved, being similar to the CON group (Figure 5A). Ammonia stress also significantly induced the mRNA levels of nuclear receptor coactivator 4 (*ncoa4*), transferrin receptor 1 (*tfr1*), *acsl4*, and *ptgs2* and decreased the mRNA levels of cellular tumor antigen (*p53*), glutathione peroxidase 4 (*gpx4*), ferroportin (*fpn*), and ferritin heavy chain 1 (*fth1*) compared with the CON group. The opposite changes were observed in the TAN + CUR group (Figure 5B,C). Meanwhile, the protein expression levels of ACSL4 and PTGS2 were significantly increased, and SLC7A11 protein expression was significantly decreased in the TAN group compared with the CON group, while contrasting changes were observed in the TAN + CUR group (Figure 5D,E). Similar results were found in fish with respect to the fluorescence intensity of ACSL4, PTGS2, and SLC7A11 (Figure 5F–I).

### 2.6. Terminal Deoxynucleotidyl Transferase dUTP Nick-End Labeling (TUNEL) Analysis

**Figure 6:**
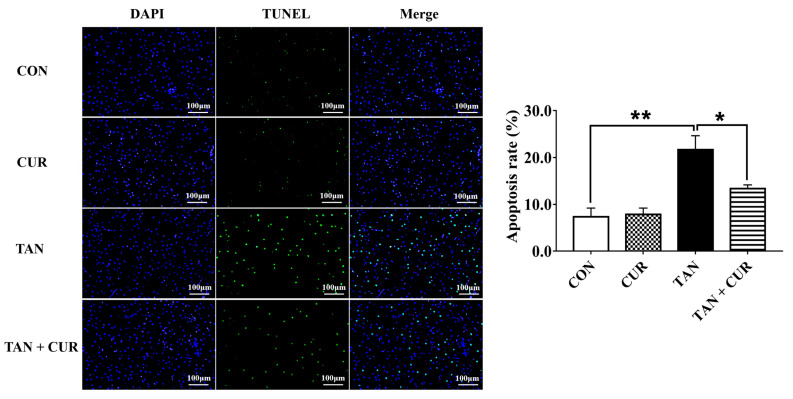
The results of terminal deoxynucleotidyl transferase dUTP nick-end labeling (TUNEL) staining in the normal and acute ammonia-stressed groups of gibel carp after eight weeks of feeding CUR diet. Under the fluorescence microscope, the apoptotic cells fluoresce in green, while the blue color represents the nucleus. Scale bar = 100 μm. The values are expressed as the mean ± standard error (*n* = 6). * *p* < 0.05; ** *p* < 0.01.

As shown in Figure 6, the apoptosis rate of hepatocytes in the TAN group was significantly higher than that in the CON group, whereas the apoptosis rate in the TAN + CUR group was significantly lower than that in the TAN group.

## 3. Discussion

CUR has wide applications in traditional medicine for its potent antioxidant and anti-inflammatory effects. As a high concentration of ammonia in the water could lead to tissue damage and high mortality in aquaculture species, CUR was hypothesized to attenuate oxidative stress and ferroptosis induced by ammonia in gibel carp. In the present study, dietary supplementation of CUR had no significant effect on SGR or FE in fish. However, it has been reported that 5 g/kg CUR supplementation significantly promoted the growth of crucian carp [20]. Similarly, dietary supplementation with 393.67 mg/kg CUR promoted growth in grass carp (*Ctenopharyngodon idella*) [21]. Thus, this discrepancy may be related to fish species, dosage, fish size, or rearing conditions.

The liver is an important organ for maintaining physiological functions. Once damaged, it will lead to metabolic disorders that can accelerate death. Ammonia stress stimulated the degeneration of carp gill tissue and the infiltration of inflammatory cells, with pronounced pyknosis of liver tissue and the presence of inflammatory cells around degenerated pancreatic cells [22]. In the present study, compared with the CON group, the liver tissue was abnormal, some hepatocytes were slightly hypertrophic, and the cytoplasm was vacuolated. Similarly, the gill tissue structure of the gibel carp in the TAN group was damaged, and some gill fragments were atrophied. The results suggested that acute ammonia stress induced pathological changes in the liver and gill. However, the histopathology of the gibel carp was significantly improved in the TAN + CUR group, confirming that CUR had a protective effect on oxidative damage induced by acute ammonia stress. ADA is a nucleic acid metabolizing enzyme that plays an important role in the cellular immune system of the body, serving as a sensitive indicator reflecting liver damage. Furthermore, ALT and AST are also considered markers of hepatocyte injury [23]. Herein, the activities of AST, ALT, and ADA in the plasma and the activities of AST and ALT in the liver were abnormally elevated in the TAN group compared with the CON group, reflecting the severe damage to the liver function of gibel carp by ammonia exposure. However, opposite changes in the levels of these indicators were observed in the TAN + CUR group, implying that CUR could effectively protect the liver from injury induced by acute ammonia stress.

Fish experience oxidative stress when stimulated by ammonia nitrogen, and this response is associated with many metabolic diseases [2]. In this study, the levels of GLU and COR in the plasma of the TAN group were significantly higher than those of the CON group after acute ammonia exposure, suggesting that ammonia initiated the stress response of the fish. However, contrasting changes were found in the TAN + CUR group, indicating that stress induced by ammonia exposure was ameliorated by CUR. Furthermore, acute ammonia stress decreased hepatic antioxidant capacity (T-AOC and SOD, GPx) in the gibel carp, while the opposite result was observed for the CUR treatment. The elevated ROS levels were mainly attributed to the disruption of the antioxidant defense barrier [24]. Impaired clearance or overproduction of lipid peroxide MDA can lead to its accumulation to lethal levels and trigger ferroptosis [25]. In our study, liver ROS and MDA levels were significantly stimulated by acute ammonia stress, similar to the results observed in yellow catfish (*Pelteobagrus fulvidraco*) [26]. Moreover, Nrf2, a key transcription factor that regulates resistance to oxidative stress, plays an essential role in the induction of antioxidant response in the organism [27]. Activation of Nrf2 signaling protected against acetaminophen-induced liver injury in mice [28]. Herein, mRNA levels of the Nrf2 pathway and its downstream antioxidant key genes (*nrf2*, *ho-1*, *cat*, and *sod*) were significantly decreased in the TAN group, suggesting that acute ammonia stress disturbed the antioxidant balance in the fish and induced oxidative stress. Oxidative stress was also induced in common carp exposed to ammonia [22]. Likewise, CUR enhanced antioxidant capacity by activating the Nrf2 pathway in the TAN + CUR group, as in *Channa argus* [7]. In addition, CUR ameliorated apoptosis induced by acute ammonia exposure in the head kidney macrophages of yellow catfish [29]. In line with this, CUR significantly enhanced the anti-apoptotic capacity in gibel carp, as indicated by inhibited apoptotic signals in the liver induced by acute ammonia stress.

In addition to autophagy and apoptosis, oxidative stress pathways can also lead to cellular ferroptosis, a non-apoptosis-regulated form of cell death [13]. It has been reported that ferroptosis was associated with a fatty liver, where the outer mitochondrial membrane was broken and shrunken in gibel carp [30]. In the present study, ferroptosis was induced in the TAN group as reflected by mitochondrial atrophy, reduction or disappearance of cristae, and increased membrane density. However, ferroptosis was significantly inhibited in the TAN + CUR group, as indicated by normal mitochondrial morphology and size. TFR1, DMT1, and FTH1 control iron uptake and storage and have important roles in maintaining intracellular iron homeostasis [31,32]. When iron homeostasis is disrupted, increased intracellular free iron promotes the production of lipid-reactive oxygen species through the Fenton reaction, promoting the onset of ferroptosis. GPX4 is a central regulator of lipid peroxidation and ferroptosis involved in the Fenton reaction [33]. A significant increase in the mRNA levels of hepatic *ncoa4* and *tfr1* and a significant decrease in the mRNA levels of *p53*, *gpx4*, *fpn*, and *fth1* were induced by ammonia stress, indicating that hepatic ferroptosis was triggered by stimulating iron uptake and reducing iron storage, eventually leading to liver injury in gibel carp, similar to the effects in yellow catfish [26]. Thus, CUR affected the process of liver injury in gibel carp through the inhibition of the ferroptosis pathway.

ACSL4 is positively correlated with ferroptosis sensitivity; it is involved in fatty acid metabolism and promotes lipid peroxide generation, a key part of ferroptosis [34]. SLC7A11 regulates the uptake of cystine, which can synthesize GSH. The decreased expression of SLC7A11 aggravates oxidative damage and promotes ferroptosis [35]. In our study, the mRNA levels of *acsl4* and *ptgs2* and the protein expression levels of ACSL4 and PTGS2 were significantly increased, and the protein expression of SLC7A11 was significantly decreased in the TAN group compared with the CON group. Similarly, the fluorescence intensity of PTGS2 and ACSL4 increased significantly, and the opposite change was observed in SLC7A11 in gibel carp exposed to acute ammonia stress. Taken together, data related to gene expression levels, protein expression levels, and fluorescence intensity in the TAN + CUR group suggested that CUR alleviates liver injury by inhibiting ferroptosis. Consistent with this hypothesis, CUR also mitigated rhabdomyolysis-related kidney damage by reducing ferroptosis in mice [36].

## 4. Materials and Methods

### 4.1. Experimental Diets

The basal diet was formulated to contain 36.83% crude protein and 6.94% crude lipid. CUR (purity: ≥95%, Beijing Solarbio Science & Technology Co. Ltd., Beijing, China) was then added at 5 g/kg according to the previous study [20]. Diet composition and nutrient ingredients are listed in Table 2. All of the ingredients were crushed and mixed well. The mixed ingredients were passed through a 40-mesh sieve, mixed with water, and then prepared into 2 mm feed pellets using a granulator (SLR-45, Fishery Machinery and Instrument Research Institute, Chinese Academy of Fishery Science, Shanghai, China). The pellets were dried in a constant temperature oven at 50 °C until the moisture content was close to 10% and then stored in a refrigerator at 4 °C.

### 4.2. Experimental Animals and Procedures

Gibel carp were obtained from the Institute of Hydrobiology, Chinese Academy of Sciences (Wuhan, China). Experimental procedures were approved by the ethics committee of the Institute of Hydrobiology, Chinese Academy of Sciences.

Before the experiment, the fish were acclimatized for at least two weeks. After that, 150 healthy fish with an average initial weight of 11.22 ± 0.10 g were randomly assigned to six tanks. Three of the tanks contained fish fed with basal diet and served as a CON group, while the rest of the fish fed diets supplemented with curcumin were considered a treatment group (CUR group). The fish were hand-fed to apparent satiation three times daily (8:00, 13:00, and 18:00) for 56 days. During the experiment, the water temperature was kept at 26.2 ± 0.2 °C; pH was 7.6 ± 0.02; total ammonia nitrogen was 0.08 ± 0.00 mg/L; nitrite was 0.03 ± 0.00 mg/L; dissolved oxygen was >7.0 mg/L, and the photoperiod was a 12 L:12 D cycle.

### 4.3. Ammonia Stress Experiments

After 56 days of culture, gibel carp were starved for 24 h and then subjected to ammonia stress (32.5 mg/L) for 24 h. The ammonia solution was prepared with 10 g/L NH_4_Cl, and the ammonia concentration in the water was determined according to a previous study [37]. The concentration of total ammonia nitrogen (TAN) was measured using a multi-parameter water quality meter (Greencarey Precision Instrument Co., Ltd., Guangzhou, China).

### 4.4. Sample Collection

Before (0 h) and after (24 h) acute ammonia exposure, two fish per tank were randomly selected and anesthetized, and thus a CON group, a CUR group, a TAN group, and a TAN + CUR group were set up. Blood samples were taken from the tail vein with a syringe soaked with heparin sodium (0.2%) and centrifuged (4000× *g*, 15 min, 4 °C) to obtain plasma. Gill and liver samples were collected and quickly fixed in paraformaldehyde (4%) for histopathological observation. Some of the remaining liver samples were put into glutaraldehyde solution (2.5%) to observe the ultrastructure, and other samples were frozen at −80 °C for subsequent biochemical detection and gene and protein expression analysis.

### 4.5. Biochemical Analysis

The composition of fish and diets were determined according to a previous study [38]. The levels of COR, GLU, LD, ALT, AST, ADA, and ALP in plasma; the activity of MDA; and the content of T-AOC, SOD, and GPx in the liver were determined with commercial kits from Nanjing Jiancheng Bioengineering Institute (Nanjing, China). The level of ROS in the liver was measured with an enzyme linked immunosorbent assay (ELISA) kit (Wuhan MSK Biological Technology Co., Ltd., Wuhan, China).

### 4.6. Histopathology

The fixed liver tissues were dehydrated with graded ethanol and were made transparent with xylene. Then, the tissues were dipped in wax and placed in an embedding machine for slicing into 4 μm thick sections using a paraffin microtome. The sections were stained with hematoxylin and eosin (H&E), making nuclei appear blue and cytoplasm appear red. Finally, the sections were observed under an optical microscope, and three representative images per section were randomly selected. For more details on H&E staining, refer to our previous study [39].

### 4.7. TUNEL Assay

A TUNEL assay was employed on liver tissue samples fixed by paraformaldehyde (4%) and embedded in paraffin. The liver samples were cut into 5 μm sections to be prepared and infiltrated, then incubated with TdT and dUTP. The sections were stained with DAPI and observed under a microscope. Image-Pro Plus 4.1 software was used for analysis. More details concerning TUNEL analysis can be found in a previous study [40].

### 4.8. TEM

The liver samples fixed in glutaraldehyde (2.5%) were trimmed into 1 mm^3^ small cubes and rinsed three times with PBS (0.1 M, pH = 7.0) for 15 min each time. After being fixed with osmium acid solution (1%) for 1 or 2 h and rinsed, the samples were dehydrated with graded ethanol (50%, 70%, 80%, 90%, and 95%) and treated with pure ethanol and acetone for 20 min. After being embedded using an embedding machine and kept at a constant temperature of 60°C overnight, the samples were cut into sections of 70–90 nm thickness with an ultrathin slicer. The sections were stained with lead citrate solution and uranyl acetate dissolved in 50% ethanol solution and examined under an HT7700 TEM (Hitachi High-Tech, Tokyo, Japan).

### 4.9. Quantitative Real-Time Polymerase Chain Reaction (qRT-PCR)

Total RNA extraction was performed according to the manufacturer’s protocol. A reverse transcription kit (M-MLV First-Strand Synthesis System) was used for cDNA synthesis. SYBR green (Roche, Germany) and a LightCycle 480 II (Roche, Germany) were used for qRT-PCR. More details are given in a previous study [37]. The gene *ef-1α* was used as the internal reference gene. Table 3 lists the relevant primers.

### 4.10. Western Blot Analysis

Western blot analysis was conducted according to a previous study [41]. The weighed liver samples were added to an equal proportion of RIPA lysis buffer (Beyotime Biotechnology, Shanghai, China), and the supernatant was obtained after ultrasonic crushing and centrifugation. A BCA kit (Beyotime Biotechnology, Shanghai, China) was used for protein concentration measurement. Then, protein samples were separated by SDS-PAGE, transferred to a membrane, and sealed. The membrane was incubated with a specific primary antibody of PTGS2, ACSL4, or SLC7A11 (Abclonal, Wuhan, China) or GAPDH (Cell Signaling Technology, Danvers, MA, USA) at 4 °C overnight. Fluorescent signals were detected with an imager (Chemidoc-MP imaging system, BioRad, Hercules, CA, USA), and the protein quantification results were generated by Image J 1.53a software (National Institutes of Health, Bethesda, MD, USA).

### 4.11. Immunofluorescence Analysis

The liver sections were successively soaked in xylene, absolute ethanol, 85% alcohol, and 75% alcohol for dewaxing. Then, the sections for antigen repair and blocking were developed. Following that, the sections were incubated with the PTGS2, ACSL4, and SLC7A11 antibodies at 4 °C overnight and with the secondary antibody goat anti-rabbit IgG H&L (HRP) (ab205718, Abcam, Cambridge, UK) at room temperature for 50 min. The nuclei were stained with DAPI. The sections were observed under a fluorescence microscope.

### 4.12. Statistical Analysis

SPSS version 19.0 (SPSS Inc., Chicago, IL, USA) was used for statistical analysis. Comparisons of the two groups were performed by one-way ANOVA. *p* < 0.05 showed a statistically significant difference, and *p* < 0.01 showed a very significant difference. Results were expressed as mean ± SEM.

## 5. Conclusions

In conclusion, our study demonstrated that CUR attenuated oxidative stress induced by ammonia via inhibiting ROS and MDA levels and by activating the Nrf2 pathway. In addition, ferroptosis triggered by acute ammonia stress was inhibited by mediating ACSL4, PTGS2, and SLC7A11 protein levels. Our data demonstrate for the first time that CUR has antagonistic effects on acute ammonia stress-induced oxidative stress and ferroptosis in gibel carp. Therefore, CUR is a potential additive for the prevention of liver injury in fish.

## Figures and Tables

**Table 1:** Growth performance and whole fish composition of gibel carp after eight weeks of Curcumin (CUR) supplementation in the diet.

Category	Parameter	Treatment	
		CON	CUR
Growth performance	W_0_^1^ (g)	11.23 ± 0.07	11.20 ± 0.10
	W_t_^2^ (g)	36.43 ± 2.29	37.63 ± 3.13
	FR ^3^ (%)	3.12 ± 0.05 ^a^	3.13 ± 0.01 ^b^
	FE ^4^ (%)	61.02 ± 0.97	60.42 ± 1.89
	SGR ^5^ (%/d)	2.10 ± 0.06	2.16 ± 0.09
Body composition	Crude protein (%)	14.62 ± 0.22	14.41 ± 0.28
	Crude lipid (%)	6.34 ± 0.17	6.13 ± 0.23
	Ash (%)	3.33 ± 0.32 ^a^	3.45 ± 0.33 ^b^
	Moisture (%)	72.24 ± 0.30	71.43 ± 0.43

The data listed in the table are all expressed as means ± SEM, and the superscripts of different letters in the same row indicate significant differences (*p* < 0.05). ^1^ Initial weight (W_0_, g) = total initial weight (g)/total number of fish; ^2^ Final weight (W_t_, g) = total final weight (g)/total number of fish;.^3^ Feeding rate (FR, % BW/d) = 100 × dry feed intake/[days × (W_0_ + W_t_)/2]; ^4^ Feed efficiency (FE, %) = (100 × fresh body weight gain)/dry feed intake; ^5^ Specific growth rate (SGR, %/d) = 100 × [ln (final weight)−ln (initial weight)]/day.

**Table 2:** Formulation and approximate composition (% dry matter) of practical diets supplemented with curcumin (CUR).

Ingredients	CON	CUR
White fish meal ^1^	15	15
Rapeseed meal ^2^	20	20
Soybean meal ^3^	25	25
Wheat flour ^4^	25.4	25.4
Oil mixture ^5^	5.5	5.5
CUR	0	0.5
Vitamin premix ^6^	0.39	0.39
Choline chloride	0.11	0.11
Mineral premix ^7^	5	5
Carboxy methyl cellulose sodium	3	3
Cellulose	0.60	0.1
Proximate chemical composition (%)		
Crude protein (%)	36.83	36.95
Crude lipid (%)	6.94	7.01
Nitrogen free extract (%)	19.96	21.48
Gross energy (kJ/g)	18.64	18.58
Moisture (%)	9.90	9.59
Ash (%)	6.58	6.43

^1^ White fish meal: Purchased from American Seafood Company, Seattle, WA, USA. Crude protein: 75.63%; Crude lipid: 4.08%. ^2^ Soybean meal: Purchased from Coland Feed Co., Ltd., Wuhan, China. Crude protein: 55.22%; Crude lipid: 1.34%. ^3^ Rapeseed meal: Purchased from Coland Feed Co., Ltd., Wuhan, China. Crude protein: 42.81%; Crude lipid: 2.02%. ^4^ Wheat flour: Crude protein: 12.64%; Crude lipid: 1.60%. ^5^ Oil mixture: soybean oil: fish oil = 1: 1. ^6^ Vitamin premix (mg kg^−1^ diet): Vitamin B_1_, 20; Vitamin B_2_, 20; Vitamin B_6_, 20; Vitamin B_12_, 0.02; folic acid, 5; calcium pantothenate, 50; inositol, 100; niacin, 100; biotin, 0.1; cellulose, 3522; Vitamin C, 100; Vitamin A, 110; Vitamin D, 20; Vitamin E, 50; Vitamin K, 10. ^7^ Mineral salt premix (mg kg^−1^ diet): NaCl, 500.0; MgSO_4_·7H_2_O, 8155.6; NaH_2_PO_4_·2H_2_O, 12,500.0; KH_2_PO_4_, 16,000; Ca(H_2_PO_4_)·2H_2_O, 7650.6; FeSO_4_·7H_2_O, 2286.2; C_6_H_10_CaO_6_·5H_2_O, 1750.0; ZnSO_4_·7H_2_O, 178.0; MnSO_4_·H_2_O, 61.4; CuSO_4_·5H_2_O, 15.5; CoSO_4_·7H_2_O, 0.91; KI, 1.5; Na_2_SeO_3_, 0.60; Corn starch, 899.7.

**Table 3:** Sequences of primers applied for quantitative real-time PCR analysis in gibel carp.

Gene Name	Sense and Antisense Primer (5′-3′)	Gene Bank	Product Length
		Accession No.	(bp)
Elongation factor 1 alpha (*ef1α*)	GTTGGAGTCAACAAGATGGACTCCAC	AB056104	198
	CTTCCATCCCTTGAACCAGCCCAT		
Nuclear factor [erythroid-derived 2]-like 2 (*nrf2*)	CCCTTCACCAAAGACAAGCA	MG759384	128
	TTGAAGTCATCCACAGGCAG		
Kelch-like ECH-associated protein-1 (*keap1*)	CTCACCCCCAACTTCCTGCAG	MG759382	150
	GATGAGCTGCGGCACCTTGGG		
Heme oxygenase-1 (*ho-1*)	GACAGGAGCATCTACCCACAG	KC758864.1	113
	GTGGCTGCTTTTATCTGCTCG		
NADPH quinine oxidoreductase-1 (*nqo-1*)	AGCAACAGAGACAACGGCAC	XM_026268231.1	176
	GTGTGCACCAGTACAGAGGAG		
Catalase (*cat*)	CTCCAACGGCAACTTCCCAT	JX477239.1	102
	CACACCTTAGTCAAATCAAA		
Superoxide dismutase (*sod*)	GTCCGCACTACAACCCTCAT	JQ776518.1	134
	GGTCACCATTTTATCCACAA		
Glutathione peroxidase (*gpx*)	GCCCACCCTCTGTTTGTGTT	DQ983598.1	244
	CAGGTTTATTTCGCCCTCTTC		
Nuclear receptor coactivator 4 (*ncoa4*)	TTTTGCCAGCGATGAAGCAC	XM_026224501.1	198
	GCCACTTCTCTTTGTCCCCA		
Transferrin receptor 1 (*tfr1*)	TATCCCAGAGTGACCGTAG	XM_026237902.1	148
	TTGTTGGGTTTGGACTTGT		
Divalent metal transporter 1 (*dmt1*)	GCTGAACTCGTCTGATCTGGAA	XM_026240820.1	296
	CCTCGAAGTATGTGGAGATGGG		
Iron-responsive element-binding protein 2 (*ireb2*)	TTGGTGCTGGATGCGAAGA	XM_026202373.1	99
	GCTCCATCAAATTGGCCGT		
Cellular tumor antigen (*p53*)	GTGCAAGATAGTCAAGCTGGTG	XM_026210616.1	245
	CTGGGGTTCTCTCATGGTGG		
Glutathione peroxidase 4 (*gpx4*)	ATCTAATACGCTGCCTGCCG	XR_003278905.1	211
	TGATGACAACTTTGCCCCTG		
Ferroportin (*fpn*)	CCTCGGACATGCTCTGTCAA	XM_026271430.1	112
	CAGTCCATACACGGCTGTCA		
Ferritin heavy chain 1 (*fth1*)	TTGCGAAGTTTTTCCGCCATC	XM_026216156.1	94
	AGAAAGATCCTCCCTCCCCTC		
Apoptosis-inducing factor mitochondria-associated 2 (*aifm2*)	GTTCAAGCGGGTTTTGCGA	XM_026224404.1	216
	GGCTGCTTGGTAGGTGTCC		
Prostaglandin-endoperoxide synthase 2 (*ptgs2*)	AAAAACATCGCCTCCTGCAAC	JX183400.1	178
	CACTGACTGGAAAGGGGAGAC		
Long chain acyl CoA synthetase 4 (*acsl4*)	ACAGTATTTATAGCAGTGAGGT	NC_039256.1	173
	ATAATAATGTTTGCATGTCCGT		

## Data Availability

Data are contained within the article.

## Supplementary Materials

The following supporting information can be downloaded at: https://www.mdpi.com/article/10.3390/ijms24076441/s1.
