# Supplementary material for: Effects of Curcumin on Oxidative Stress and Ferroptosis in Acute Ammonia Stress-Induced Liver Injury in Gibel Carp (Carassius gibelio)
Source: Int J Mol Sci. 2023 Mar 29;24(7):6441. doi: 10.3390/ijms24076441 (PMC10094298; doi:10.3390/ijms24076441)
Supplement: Supplementary file 1 [file ijms-24-06441-s001.zip › ijms-2271103-supplementary.pdf]

Effects of Curcumin on Oxidative Stress and Ferroptosis in Acute Ammonia Stress-Induced Liver Injury in Gibel Carp (*Carassius gibelio*)

Liyun Wu [1,2], Bo Dong [1,2], Qiaozhen Chen [1,2], Yu Wang [1,2], Dong Han [1,2], Xiaoming Zhu [1], Haokun Liu [1], Zhimin Zhang [1], Yunxia Yang [1], Shouqi Xie [1,2,3] and Junyan Jin [1,*]

1 State Key Laboratory of Freshwater Ecology and Biotechnology, Institute of Hydrobiology, Chinese Academy of Sciences, Wuhan 430072, China

2 University of Chinese Academy of Sciences, Beijing 100049, China

3 The Innovative Academy of Seed Design, Chinese Academy of Sciences, Beijing 100101, China

[*]*Correspondence: Junyan Jin*

E-mail address: jinjunyan@ihb.ac.cn

Table S1. Table of raw data of plasma and liver metabolites of the gibel carp.

Items		CON Group						CUR Group					TAN Group						TAN + CUR Group						
		**1**	**2**	**3**	**4**	**5**	**6**	**1**	**2**	**3**	**4**	**5**	**6**	**1**	**2**	**3**	**4**	**5**	**6**	**1**	**2**	**3**	**4**	**5**	**6**
**Plasma metabolites**	GLU (mmol/L)	4.4	4.6	5.1	5.7	6.5	8.4	3.7	5.3	3.0	6.5	6.1	5.4	37.5	22.3	38.4	11.3	49.7	17.4	13.0	10.5	8.8	9.6	13.6	10.1
	COR (ng/mL)	76.6	72.6	53.0	77.3	74.0	87.2	80.6	37.8	80.2	40.7	52.6	27.6	122.3	82.9	100.1	93.1	83.1	114.6	64.5	36.1	66.2	59.5	87.5	69.8
	LD (mmol/L)	3.6	4.3	4.4	4.0	3.9	3.9	4.8	5.2	4.2	5.5	5.7	5.7	7.4	2.7	2.8	2.8	3.8	2.8	1.4	2.1	2.9	4.8	3.3	1.9
	AST (U/L)	18.9	22.2	21.6	18.1	15.2	21.9	18.9	23.6	31.6	16.0	20.0	22.7	27.0	31.0	23.0	29.6	39.0	33.7	26.4	21.6	28.7	21.1	23.9	23.9
	ALT (U/L)	3.2	1.2	17.9	1.9	2.8	1.7	8.8	9.3	0.8	1.7	2.8	1.1	12.7	8.4	10.2	0.5	6.5	11.2	3.4	5.2	4.4	0.0	7.4	5.7
	ADA (U/L)	25.1	20.3	31.8	35.7	14.5	31.8	35.7	33.8	13.5	29.9	38.6	32.8	32.8	30.9	57.9	34.7	41.5	44.4	30.9	28.0	30.9	32.8	11.6	24.1
	ALP (King/L)	53.9	52.1	52.1	50.3	48.5	44.9	41.3	55.7	43.1	55.7	43.1	43.1	52.1	48.5	66.5	73.7	70.1	77.2	62.9	57.5	62.9	57.5	62.9	52.1
**Liver metabolites**	ROS (ng/mg prot)	0.6	0.1	0.1	0.1	0.2	0.1	0.1	1.1	0.3	0.2	0.2	0.2	0.4	0.6	0.7	0.4	0.7	0.4	0.2	0.1	0.1	0.2	0.1	0.1
	MDA (nmol/mg prot)	0.5	0.6	0.7	0.5	0.6	0.4	0.5	0.9	0.4	0.5	0.6	0.6	1.4	0.6	0.7	1.1	1.8	0.6	0.7	0.4	0.4	0.5	0.5	0.5
	T-AOC (nmol/mg prot)	0.2	0.2	0.2	0.2	0.2	0.2	0.3	0.2	0.4	0.3	0.3	0.3	0.1	0.1	0.1	0.1	0.0	0.1	0.0	0.4	0.2	0.2	0.1	0.1
	SOD (U/mg prot)	381.5	401.4	411.5	387.0	441.0	409.4	407.4	457.2	495.8	0.0	499.3	535.7	152.6	308.2	319.5	229.6	190.5	192.8	418.3	387.9	106.7	387.8	382.3	240.1
	GPx (U/mg prot)	983.4	1103.8	998.4	928.2	893.8	1153.8	1094.5	1057.9	692.5	930.7	979.1	1086.6	899.2	653.5	620.9	754.8	679.2	400.6	793.8	880.0	828.7	804.7	799.0	796.0
	AST (U/g prot)	12.2	13.0	12.6	13.2	13.2	14.0	14.2	14.4	18.5	13.7	14.4	14.8	12.9	18.3	19.4	15.7	14.7	14.7	7.6	8.9	8.2	8.6	8.5	8.9
	ALT (U/g prot)	32.6	32.8	33.7	34.9	39.1	35.2	37.8	31.7	37.1	36.9	36.3	38.0	42.3	45.4	40.9	39.0	0.0	40.0	43.4	37.2	35.8	38.3	32.1	41.5
